# The impact of funding for federally qualified health centers on utilization and emergency department visits in Massachusetts

**DOI:** 10.1371/journal.pone.0243279

**Published:** 2020-12-03

**Authors:** Catherine Myong, Peter Hull, Mary Price, John Hsu, Joseph P. Newhouse, Vicki Fung

**Affiliations:** 1 Health Policy Research Center, Mongan Institute, Massachusetts General Hospital, Boston, Massachusetts, United States of America; 2 The Becker Friedman Institute, University of Chicago, Chicago, Illinois, United States of America; 3 Department of Medicine, Harvard Medical School, Boston, Massachusetts, United States of America; 4 Department of Health Care Policy, Harvard Medical School, Boston, Massachusetts, United States of America; 5 Department of Health Policy and Management, Harvard T.H. Chan School of Public Health, Boston, Massachusetts, United States of America; 6 Harvard Kennedy School, Cambridge, Massachusetts, United States of America; Auburn University, UNITED STATES

## Abstract

**Importance:**

Federally qualified health centers (FQHCs) receive federal funding to serve medically underserved areas and provide a range of services including comprehensive primary care, enabling services, and behavioral health care. Greater funding for FQHCs could increase the local availability of clinic-based care and help reduce more costly resource use, such as emergency department visits (ED).

**Objective:**

To examine the impact of funding increases for FQHCs after the ACA on the use of FQHCs and EDs.

**Methods:**

Retrospective study using the Massachusetts All Payer Claims Database (APCD) 2010–2013 that included APCD enrollees in 559 Massachusetts ZIP codes (N = 6,173,563 in 2010). We calculated shift-share predictions of changes in FQHC funding at the ZIP code-level for FQHCs that received Community Health Center funds in any year, 2010–13 (N = 31). Outcomes were the number of ZIP code enrollees with visits to FQHCs and EDs, overall and for emergent and non-emergent diagnoses.

**Results:**

In 2010, 4% of study subjects visited a FQHC, and they were more likely to be younger, have Medicaid, and live in low-income areas. We found that a standard deviation increase in prior year FQHC funding (+31 percentage point (pp)) at the ZIP code level was associated with a 2.3pp (95% CI 0.7pp to 3.8pp) increase in enrollees with FQHC visits and a 1.3pp (95% CI -2.3pp to -0.3pp) decrease in enrollees with non-emergent ED visits, but no significant change in emergent ED visits (0.3pp, 95% CI -0.8pp to 1.4pp).

**Conclusions:**

We found that areas exposed to greater FQHC funding increases had more growth in the number of enrollees seen by FQHCs and greater reductions in ED visits for non-emergent conditions. Investment in FQHCs could be a promising approach to increase access to care for underserved populations and reduce costly ED visits, especially for primary care treatable or non-emergent conditions.

## Introduction

The Patient Protection and Affordable Care Act (ACA) provided $11 billion in new funding from 2011 to 2015 to federally qualified health centers (FQHCs) through creation of the Community Health Center Fund (CHCF). FQHCs are safety net providers that qualify for federal funding from the Health Resources and Services Administration (HRSA) Health Center program to provide comprehensive primary care and serve medically underserved areas or populations regardless of ability to pay. The increases in funding for FQHCs was intended to boost the availability of primary care and help accommodate potential increases in demand for care from ACA-related coverage expansion for low-income individuals. Between 2010 and 2016, annual federal funding for FQHCs grew from $2.8 to $4.7 billion, accompanied by a $2.1 to $3.3 billion increase in funding from non-federal sources.

Increases in FQHC funding could improve access to primary care, especially for lower-income populations without a usual source of care. Prior studies have shown that increases in FQHC federal funding are associated with growth in number of patients, visits, delivery sites, and scope of services at FQHCs, both before and after passage of the ACA [1–3]. Lower-income adults and children residing in areas with more FQHCs or greater FQHC funding increases have also been found to be more likely to have office visits [4, 5].

Whether such improvements in access have downstream effects, such as reducing low value use of the emergency department is less clear. The existing evidence on FQHC use and ED visits is largely based on cross-sectional comparisons and has mixed findings [6–9]. One county-level study in California that examined the impact of expanded FQHC capacity found that increased geographic density of FQHCs reduced ED visits for uninsured residents, but not Medicaid enrollees; other measures of local FQHC capacity were not associated with changes in ED visits [10].

Clarifying the impact of funding changes for FQHCs on local care patterns is important for ongoing policy decisions. Since its creation in 2010, the CHCF has grown to be the primary source of federal funding for FQHCs; in 2017, it represented 72% of federal health center funding [11]. The fund faced difficult reauthorizations in Congress in 2015 and 2018, and a recent short-term extension of the CHCF will expire on December 11, 2020 [12–15]. Although multiple bills were introduced in the 116^th^ Congress that would extend the CHCF for an additional five years, none of them have passed yet [16].

To assess the impact of changes in FQHC funding on local FQHC visits and ED visits, we used longitudinal data from the Massachusetts All Payer Claims Database (APCD) from 2010–2013 and leveraged the substantial funding changes since the passage of the ACA.

## Materials and methods

### Data sources and population

The data sources for this study are the Massachusetts APCD version 3 and the Uniform Data System (UDS) with data on FQHCs from HRSA. The APCD v3 includes individual-level data from 2009–2013 medical claims for Massachusetts residents. Because the APCD only includes care billed to insurers, it does not include information on care received by the uninsured. The file available to researchers also excludes claims for Medicare fee-for-service beneficiaries, thus, the study population is limited to individuals with Medicaid, Medicare Advantage, or commercial coverage in 559 Massachusetts ZIP codes (N = 6,173,563 in 2010). Although the uninsured comprise a disproportionate share of FQHC patients, the uninsured rate in Massachusetts remained below 4% throughout the study period, lower than in any other state due to health reform efforts in 2006. Another advantage of studying the Massachusetts population is that Massachusetts had relatively generous Medicaid eligibility rules prior to ACA Medicaid expansion, so we are better able to isolate the effect of the FQHC funding increases from other ACA policy changes.

The UDS contains FQHC-level data on annual funding, number of patients served by ZIP code, and other aggregate patient characteristics, including sociodemographics, insurance type, and types of services received. Our analysis included 31 FQHCs in Massachusetts that received grants from the CHCF in 2010–2013 and excluded the 5 community health centers that solely received funds for special populations (e.g. migrants, homeless). This study was approved by the Mass General Brigham Institutional Review Board for research related to secondary use of data, including a waiver of consent.

### Predicted funding changes

The prior literature that examines area-level effects of FQHC availability or expansions have used a range of catchment areas for FQHCs, including county, metropolitan statistical area, and hospital referral region [4, 5, 10]. However, the actual service areas of FQHCs depend on the number, size, and geographic spread of each FQHC’s delivery sites and the local availability of non-FQHC primary care providers; the actual service areas could cross boundaries of predefined catchment areas. To address these challenges, we used information from the UDS on the number of patients each FQHC served by ZIP code to construct shift-share predictions of aggregate changes in FQHC funding for each ZIP code in Massachusetts.

**Fig 1:**
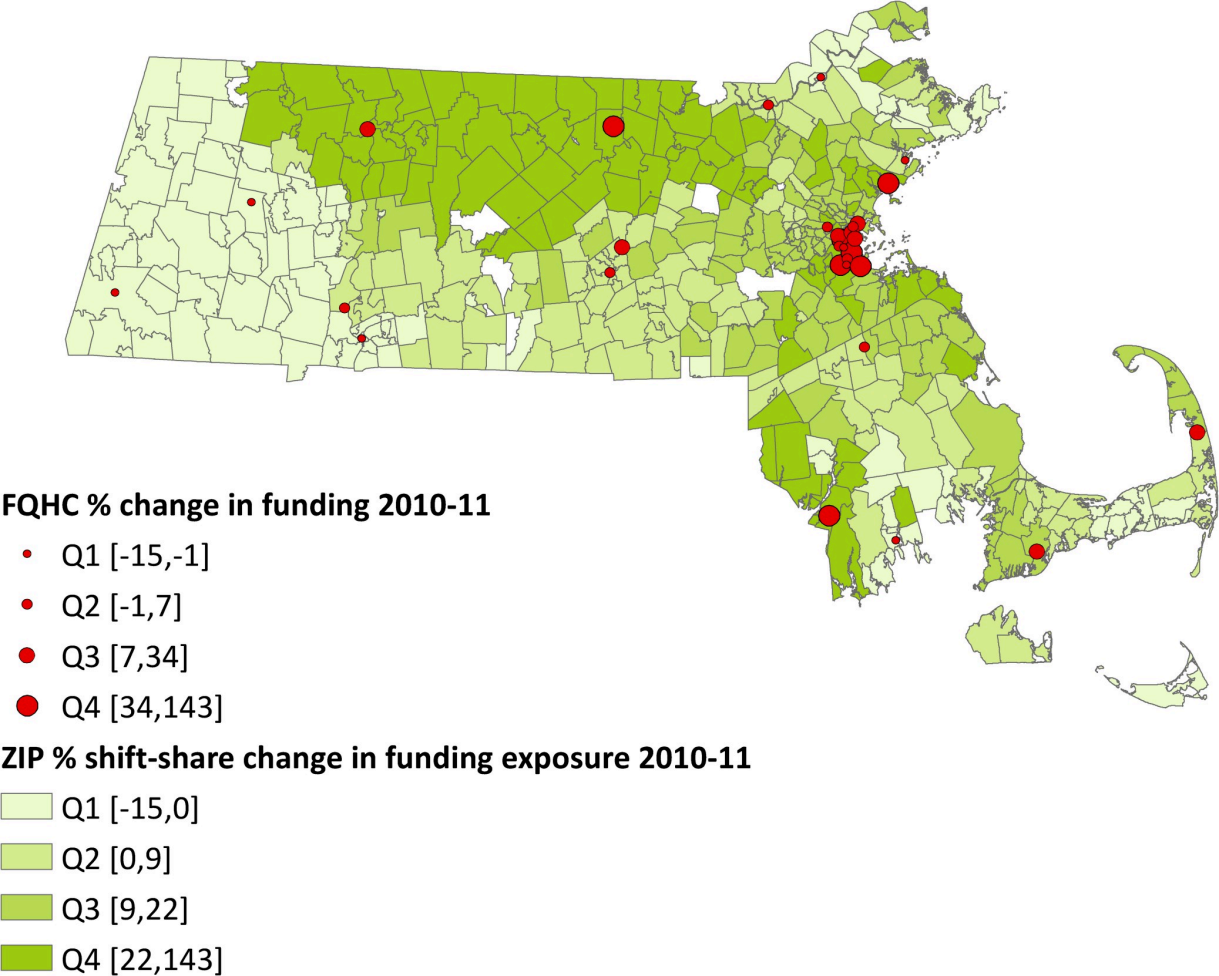
Estimated ZIP code exposure to FQHC funding changes using shift-share approach (2010–2011). Note: Q1 = 1^st^ Quartile, Q2 = 2^nd^ Quartile, etc. which range between the numbers in brackets. Red circles represent FQHCs in their geographic locations, with the size of the circle corresponding to the magnitude of the change in funding received by that FQHC from 2010–2011. Five-digit ZIP code areas are colored by shades of green, with darker areas indicating ZIP codes that were exposed to larger changes in FQHC funding between 2010–2011. Map data from U.S. Census Bureau TIGER/Line® Shapefiles (https://www2.census.gov/geo/tiger/TIGER2017/) in the public domain. Data from the Uniform Data System (UDS) and the Massachusetts All-Payer Claims Database (APCD) (2010–2011).

The shift-share approach was developed by economists to estimate local labor market effects of national industry demand shocks, though they have since been used in other settings [17–19]. Borusyak et al. (2018) demonstrates how shift-share design uses idiosyncratic variation in industry-level changes to identify causal effects that manifest in local markets. Using the shift-share approach, we used variation across ZIP codes in predicted exposure to changes in FQHC funding to identify the effects of funding changes on outcomes. To estimate predicted exposure to FQHC funding changes, we used the UDS to calculate the annual percentage change in total federal and non-federal funding for individual FQHCs. Then we estimated funding changes at the FQHC*ZIP code level by applying weights that approximate the share of funding that each FQHC allocated to the given ZIP code in 2009, the year prior to our study period. Since data on how FQHC distribute their funding across their clinic sites or service areas are unavailable, we use UDS data on the number of patients in each ZIP code who visited each FQHC in 2009 and FQHC funding levels in 2009, to impute weights under the assumption that FQHCs allocate funds across ZIP codes proportional to patient demand. Finally, because multiple FQHCs can serve the same ZIP code, we summed predicted funding changes within each ZIP code across FQHCs that had patients in those ZIP codes in 2009. For the average ZIP code, the predictor averaged funding changes across 11 FQHCs (range: 0–31). Fig 1 presents the shift-share predictions for 559 ZIP codes in 2010–2011, the location of 31 FQHCs, and the magnitude of funding change at each FQHC for comparison with our constructed predictor (see S1 Fig for 2011–2013 estimates). Thirty-two ZIP codes had no FQHC users throughout the study period. We include a detailed description of the shift-share design in S1 Appendix.

A key requirement of this shift-share design is that funding changes are quasi-randomly distributed across FQHCs, as if by a natural policy experiment. A concern is that some FQHCs could obtain funds more or less successfully due to endogenous characteristics of populations they serve and the demand for FQHC care. The heterogeneity in the types of grants HRSA awards to FQHCs helps to mitigate this concern. For example, FQHCs can obtain grants for new access points, capital investments, or health information technology development, as well as receive regular, annual appropriations to health centers under Section 330 [20]. Nevertheless, to test the assumption of quasi-random distribution of funding changes across health centers, we regressed the annual percentage change in FQHC funding levels on various observable FQHC traits, including number of patients and patient demographics (e.g., race/ethnicity, insurance type). We did not find significant associations between FQHC characteristics and annual percent funding changes, which supported this key assumption, although we cannot rule out unobservable differences across FQHCs with larger vs. smaller funding changes (see S1 Table).

### Outcome variables

Our primary outcome of change in FQHC use was the annual percentage change (2010–11, 2011–12, 2012–13) in the number of unique people in a ZIP code who made a visit to any FQHC based on medical claims in the APCD. We identified outpatient claims in the APCD with a service or billing National Provider Identifier (NPI) that corresponded to an FQHC name or address. We compared the number of unique Medicaid patients who visited each FQHC based on APCD claims with the number of Medicaid patients reported by FQHCs in the UDS and found a high correlation of 0.87 (see S2 Fig).

Our primary outcomes of change in ED use was the annual percentage change in the number of unique people with ED visits in a ZIP code overall and for emergent and non-emergent visits. We used the New York University Emergency Department (NYU ED) visit severity algorithm to classify emergent vs. non-emergent ED visits using a previously validated approach [21, 22]. The NYU algorithm assigns the probability that an ED visit falls into each of four categories: 1) non-emergency; 2) emergency treatable in a primary care setting; 3) emergency, not treatable in a primary care setting, but preventable or avoidable; 4) emergency that is not preventable or avoidable.

We classified ED visits where the probability of being a non-emergency or an emergency treatable in a primary care setting was greater than 75% as non-emergent visits (40% of visits) based on a prior validation study [21]. ED visits with a probability of being an emergency not treatable in a primary care setting (either preventable/avoidable or not) greater than 75% were classified as emergent visits (13% of visits). The NYU ED algorithm separately classifies injury-related and mental health-related ED visits (24% and 5% of ED visits, respectively), and 18% of visits had an unknown visit classification. For all outcomes, we capped values at the 99^th^ percentile to limit the influence of outliers.

### Covariates

Using the APCD, we controlled for the ZIP-code level age distribution (i.e., % age <18, 18–39, 40–64, and 65+), and median HHS-HCC (hierarchical condition category) scores, as age and underlying comorbidities would affect health care utilization choices. HHS-HCC scores, which were developed for risk adjustment in the ACA marketplaces, summarize ICD-9 diagnosis codes from claims into HCCs to predict spending, using separate models for adult, child, and infant populations to account for clinical differences across age groups [23].

Those who are uninsured or Medicare beneficiaries are not included in the APCD, but because their distribution is likely to affect patient revenue at FQHCs, we applied the exposure weights to FQHC-level insurance mix data reported in the UDS, in order to control for proportions of FQHC patients who are uninsured or on Medicare in each ZIP code. The shift-share design allows exposure weights to be applied to FQHC-level covariates, in addition to the main predictor, to control for ZIP-level time-varying observables [17]. We similarly adjusted for the number of patients who received dental or substance use disorder services, in order to account for potential expansions in FQHC capacity not captured by medical claims in the APCD.

### Statistical analysis

We used a linear regression model to estimate the effect of year-to-year changes in funding (2010–2013) on outcomes of interest; these models included ZIP code fixed effects to estimate changes within ZIP code and account for potential unmeasured time-invariant traits at the ZIP code level. We hypothesized that FQHC funding changes may have current and lagged effects on capacity, as health centers may need time to hire and train new staff, make capital improvements, and/or build new delivery sites, and so we constructed shift-share predictions for funding changes in the same year as the changes in outcome took place, and two lagged years (one year prior and two years prior to changes in outcome).

We weighted regressions by ZIP code population in the APCD and clustered the standard errors by ZIP code. For all model results, we scaled the coefficients to represent a standard deviation change in the percentage change in funding. As a falsification test, we estimated the impact of future year funding changes (i.e., the next year funding change) on ED visits overall and by subtype (see S3 Fig).

All statistical analyses were performed using Stata 14.

### Sensitivity analysis

Secondary outcomes included changes in the number of visits to any FQHC and the total number of ED visits overall and for emergent and non-emergent visits at the ZIP-code level. For ED visit classification, we also tested an alternate probability threshold of 50% to classify emergent and non-emergent visits using the NYU ED algorithm; this threshold classified 45% of visits as non-emergent and 26% of visits as emergent (see S4 Fig). We also repeated our analysis with the Bonferroni correction for four primary outcomes.

## Results

### FQHC funding changes and visit patterns in Massachusetts

Both federal and non-federal funding for FQHCs increased in aggregate from 2010–2011 and 2011–2012, and decreased in 2012–2013 (Table 1); funding for FQHCs in Massachusetts followed a similar trend but with larger relative changes. Non-federal funding increased across all years nationally and in Massachusetts, and comprised a much larger share of funding in Massachusetts than the national average (e.g., 77% vs. 46% in 2013). The total number of Massachusetts residents with FQHC visits and ED visits included in the APCD also increased over this three-year period.

**Table 1:** Changes in FQHC funding and FQHC visit patterns, 2010–2013.

	2010	2011	% change (2010–11)	2012	% change (2011–12)	2013	% change (2012–13)
**National**							
Federal funding ($ millions)	$2,794	$3,146	12.6%	$3,210	2.0%	$3,146	-2.0%
Non-federal funding ($ millions)	$2,137	$2,239	4.8%	$2,458	9.8%	$2,648	7.7%
Total funding ($ millions)	$4,931	$5,385	9.2%	$5,667	5.2%	$5,794	2.2%
**Massachusetts**							
Federal funding ($ millions)	$84	$125	48.2%	$141	13.4%	$84	-40.6%
Non-federal funding ($ millions)	$200	$216	8.2%	$239	10.7%	$276	15.6%
Total funding ($ millions)	$284	$341	20.1%	$381	11.7%	$361	-5.3%
Total FQHC visits	1,102,871	1,134,442	2.9%	1,208,350	6.5%	1,251,766	3.6%
N people with FQHC visits	207,927	214,643	3.2%	225,876	5.2%	246,500	9.1%
ED visits	2,498,501	2,574,321	3.0%	2,642,755	2.7%	2,748,064	4.0%
N people with ED visits	1,117,682	1,142,325	2.2%	1,134,438	-0.0%	1,172,574	3.4%

Notes: Funding information obtained from the UDS; number of FQHC visits, patients with FQHC visits, and ED visits obtained from the Massachusetts APCD; N people with visits among those with medical insurance in January in each year included in the APCD.

### Characteristics of FQHC patients in Massachusetts

We estimated that about 4% of all enrollees included in the APCD data visited an FQHC in 2010. As expected, those who visited FQHCs compared to those who did not were more likely to be younger (e.g., 31% vs. 22% <18 years old), have Medicaid (67% vs. 17%) and were less likely to be commercially insured (30% vs. 73%, Table 2). In addition, those who visited FQHCs were more likely to live in ZIP codes with a greater proportion of residents with incomes below 200% of the federal poverty level (37% vs. 22%) and had slightly higher HCC scores on average (0.55 vs. 0.49).

**Table 2:** Characteristics of Massachusetts residents with at least one FQHC visit versus no visits in 2010 in APCD.

	Any FQHC visit in 2010	No FQHC visit in 2010
	N = 207,927	N = 5,099,099
**Age categories**		
<18	31.2%	22.4%
18–44	38.1%	36.0%
45–64	24.0%	28.2%
65+	6.7%	13.5%
**Gender:** Female	58.4%	52.7%
**Race**		
American Indian/Alaska Native	0.5%	0.2%
Asian	5.9%	0.9%
Black	10.0%	2.2%
Hispanic	21.3%	3.7%
White	20.0%	14.4%
Other	0.9%	3.1%
Unknown/missing	41.4%	75.4%
**Insurance type (Jan)**		
Commercial total	29.6%	72.5%
Medicare	3.0%	7.7%
Medicaid	67.1%	17.2%
Health Safety Net	0.9%	1.5%
**Median HCC score**		
Overall	0.55	0.49
<18	0.19	0.11
18+	0.80	0.58
Mean % of patients’ ZIP code with **income <200% FPL**	37.2%	22.2%

Notes: APCD enrollees included if living in Massachusetts and had medical coverage in January 2010.

Compared with the traits of FQHC patients reported in the UDS, our APCD data included fewer patients overall, and had a greater proportion of Medicaid and commercially insured, as expected (see S2 Table). Age and gender distributions were similar between the APCD sample and UDS–e.g., 31% vs. 24% of FQHC patients in the APCD vs. UDS were age 18 or younger, and 7% vs. 8% were age 65 or older.

### FQHC funding changes and FQHC visits

**Fig 2:**
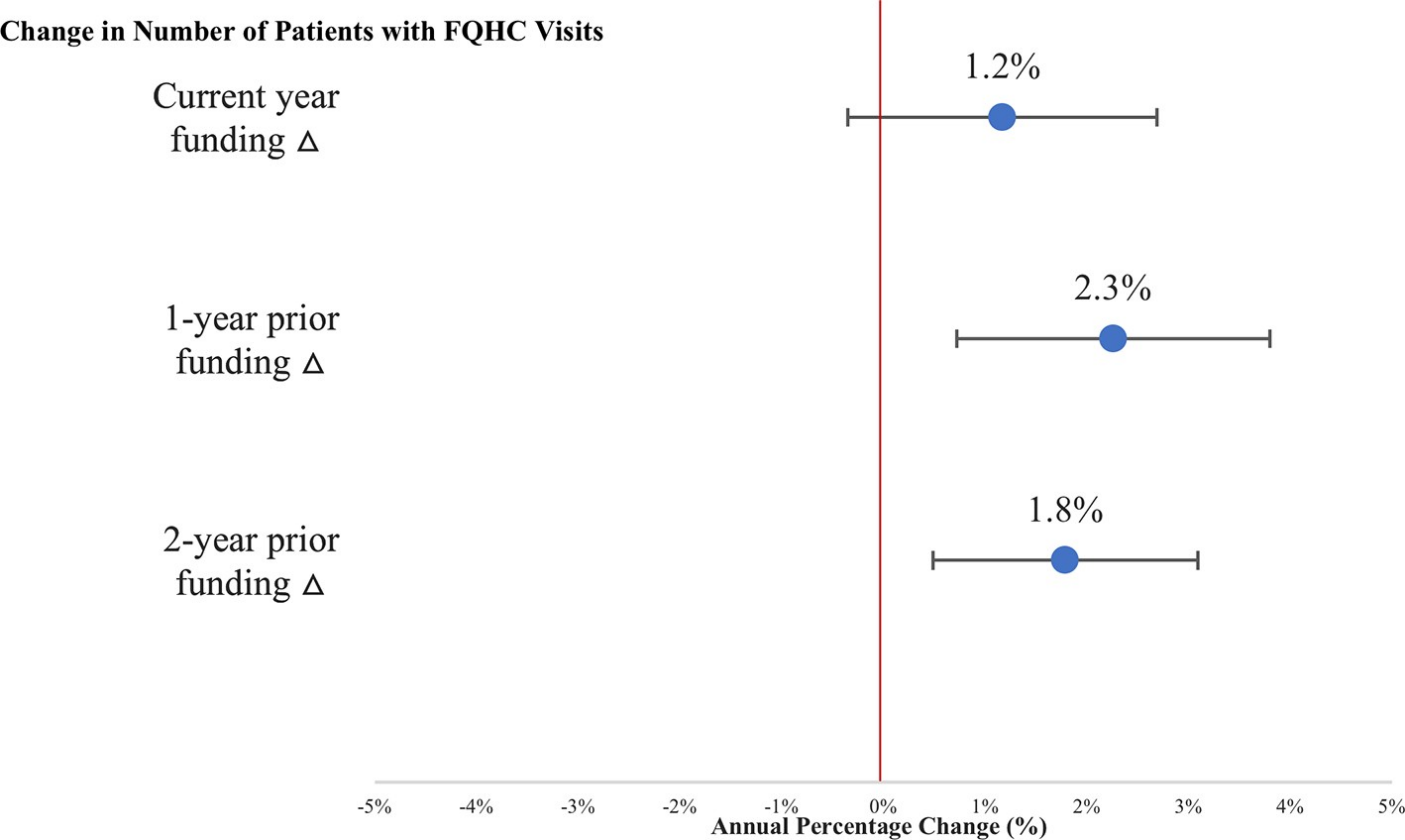
Association between changes in total funding and APCD enrollees with FQHC visits. Notes: “Current year” refers to year concurrent to change in outcome, which is one of 2010–11, 2011–12, or 2012–13. Regressions weighted by ZIP code population; outcomes capped at 99th percentile; coefficients scaled to represent change in outcomes associated with a 1 SD change in predictors (Current year % Δ all funds SD = 32.07; 1-year prior % Δ all funds SD = 31.05; 2-year prior % Δ all funds SD = 21.94). Bars indicate 95% confidence intervals.

In multivariate analyses, the number of local enrollees with FQHC visits was positively associated with one-prior year and two-prior year funding changes. For example, a one standard deviation change in prior year funding (+31 percentage point) was associated with a 2.3pp (95% CI: 0.72pp to 3.81 pp) increase in the number of enrollees in the ZIP code with an FQHC visit (Fig 2).

### FQHC funding changes and ED visits

**Fig 3:**
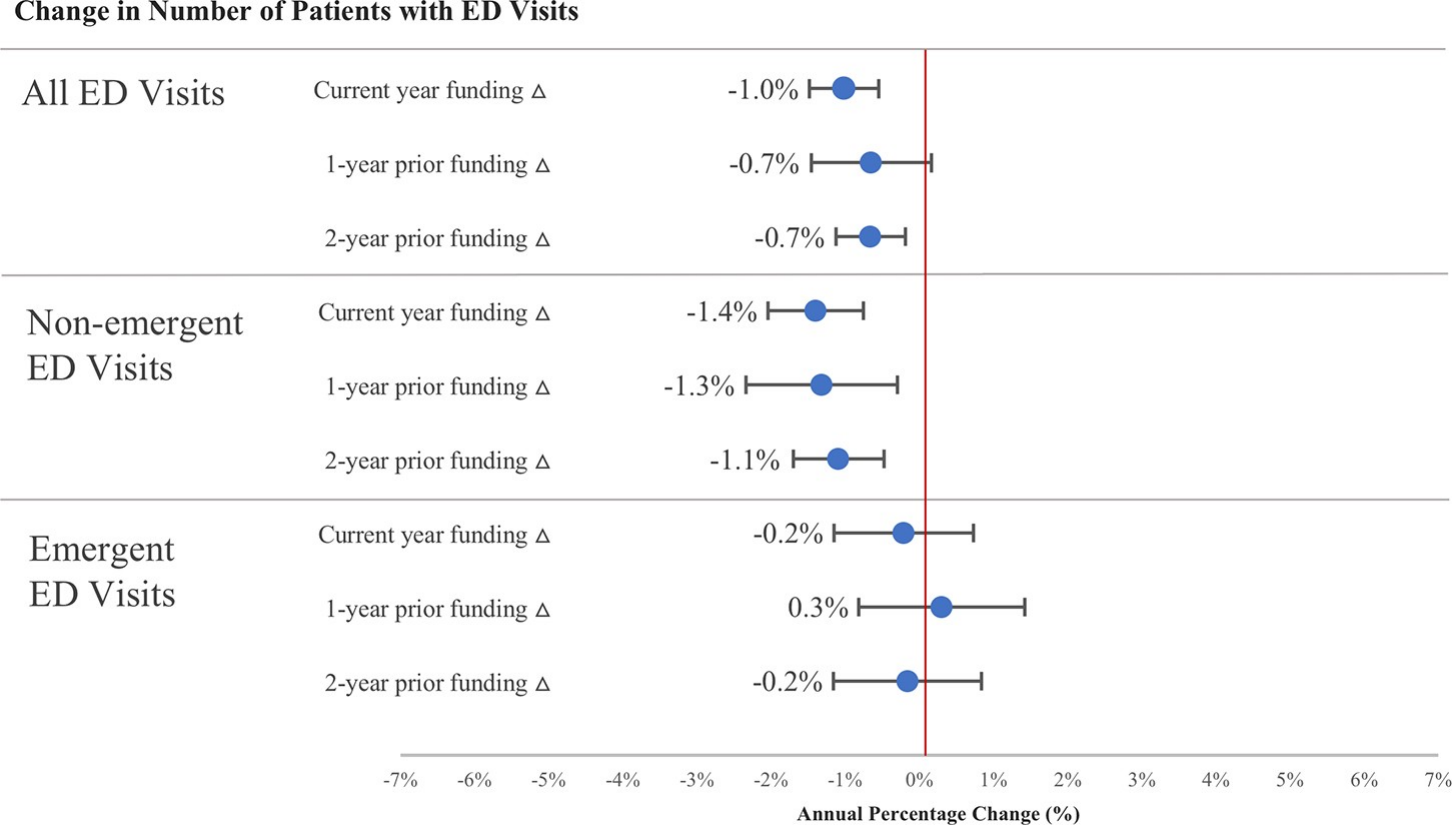
Association between changes in total funding and APCD enrollees with ED visits. Notes: “Current year” refers to year concurrent to change in outcome, which is one of 2010–11, 2011–12, or 2012–13. Regressions weighted by ZIP code population; outcomes capped at 99th percentile; coefficients scaled to represent change in outcomes associated with a 1 SD change in predictors (Current year % Δ all funds SD = 32.07; 1-year prior % Δ all funds SD = 31.05; 2-year prior % Δ all funds SD = 21.94). Bars indicate 95% confidence intervals.

Changes in funding for FQHCs in current and previous years were associated with decreases in the number of enrollees with ED visits overall (e.g., additional 32pp increase in current year funding associated with 1.0pp (95% CI: -1.5pp to -0.6pp) decrease, see Fig 3). In analyses that classified visits more likely to be non-emergent vs. emergent, we found increases in funding were associated with decreases in the number of enrollees with non-emergent visits (e.g., +32pp increase in current year funding associated with 1.4pp (95% CI: -2.0pp to -0.8pp) decrease in number of enrollees with non-emergent visits) but did not find an effect on emergent visits.

### Sensitivity analyses and falsification tests

In sensitivity analyses that examined changes in the number of FQHC visits, we did not find significant associations with funding changes, but findings for changes in the number of ED visits were similar to the primary analyses (see S5 Fig). In our falsification tests that examined the impact of future year funding changes on ED visits, we found no association between next year funding changes and ED visits overall or non-emergent visits; there was, however, a negative relationship with emergent visits. Finally, we applied the Bonferroni correction for four primary outcomes, and there were no changes in our findings (see S3 Table).

## Discussion

We examined changes in FQHC funding in Massachusetts between 2010–2013, which included the first three years of the availability of Community Health Center Funds. Over this time period there were statewide increases in FQHC funding and the number of patients receiving care from FQHCs. As expected, those who visited FQHCs in our sample lived in poorer neighborhoods and were more likely to have Medicaid insurance compared with those who did not. In analyses that exploited quasi-experimental variation in funding changes across FQHCs, we found that areas exposed to greater funding increases had more growth in the number of patients seen by FQHCs and greater reductions in the number of people with ED visits, especially for non-emergent conditions.

Prior studies of investment in FQHCs have focused on the effects of funding increases that occurred through major federal appropriations, such as the Health Center Growth Initiative in 2001, the American Recovery and Reinvestment Act of 2009, and the ACA [2–4, 24]. Few have examined how changes in total aggregate funding from all sources impact outcomes [1]. In Massachusetts, non-federal grants from state, local, and private sources comprised the majority of non-patient revenue. Nationally, nearly half of non-patient revenue for FQHCs comes from non-federal sources, underscoring the importance of accounting for these additional funding sources for FQHCs.

We found that prior year funding increases had positive, but modest effects on the total number of people who visited a FQHC; however, we did not find significant effects on visit volume. This finding could reflect heterogeneity in how FQHCs used their additional funding. For example, some might expand their visit capacity, by creating new delivery sites or improving existing sites, or offer new services, which could increase the reach of FQHCs, but not necessarily the average number of visits per patient. It is possible that FQHCs improved their visit quality, intensity, or service offerings, although we were not able to assess this directly with the available data. Funding increases could also help FQHCs provide enabling services to patients to improve health literacy or promotion or assist with non-medical needs, such as food, housing, and transportation, which would not be captured by medical claims. Importantly, many of these enabling services are not reimbursed by insurance and the availability of such services could be more susceptible to changes in funding. Lastly, these changes could take time to manifest, such as the time needed to hire and train new staff. Future research should investigate the extent to which funding changes influenced these potential mechanisms, such as by examining FQHC level changes in service availability and staffing mix.

Prior work has found that those with public vs. commercial insurance in Massachusetts were more likely to have non-emergent ED visits [25]. Consistent with these findings and our hypotheses, we found that although the total number of ED visits increased in Massachusetts from 2010 to 2013, ED visits were lower in ZIP codes with greater predicted increases in FQHC funding, especially for non-emergent visits. FQHCs could reduce ED visits by providing more underserved individuals with a usual source of care and more options for primary care treatable conditions [26]. In recent surveys, a growing proportion of FQHCs report that they are able to accommodate same or next-day appointments, and provide telephone advice outside of regular operating hours [27]. Targeting improvements in primary care availability through increases in FQHC funding could help shift visits from the ED to clinic setting for low-income populations, and reduce low-value medical spending. The average cost of a preventable ED visit in Massachusetts was about $500 in 2010 [28] and it is possible that cost savings associated with reductions in ED visits found in our study are modest compared with the changes in FQHC funding. However, ED visits represent just one potential change in direct (or indirect) costs associated with greater availability of FQHC care; for example, other studies have found differences in hospitalization rates associated with FQHC use [29].

### Limitations

This is a non-randomized study; although we found little evidence that FQHC funding changes correlate with observable characteristics, and control for characteristics of both FQHCs and ZIP codes in analysis, we cannot rule out the potential for unmeasured confounding. We included ZIP code fixed effects to account for time-invariant differences across ZIP codes; if there were changes in ZIP code level traits, such as socioeconomic status, between 2010–2013, this could confound our findings. Our area-level analysis assesses how changes in exposure to FQHC funding changes impacts local changes in proportion of residents with FQHC visits and ED visits, and inferences should not be made at the individual-level.

The APCD does not include claims for the uninsured or Medicare fee-for-service enrollees (inclusive of dual-eligibles), who were 82% of all Medicare beneficiaries in Massachusetts in 2013 [30]. According to UDS data on insurance types aggregated across the 31 FQHCs included in our study, 11% of patients were Medicare beneficiaries and 17% were uninsured in 2013. We adjusted at the center level for the annual percentage of FQHC patients covered by Medicare or uninsured in our models to address this limitation, but bias could remain if funding differentially affects care patterns across insurance coverage types.

Although our patient counts in the APCD were highly correlated with patient counts in the UDS at the FQHC level, we identified fewer people in our sample overall that used FQHCs compared with the number of patients reported in the UDS. For example, in 2010, the 31 study FQHCs in Massachusetts reported a total of about 347,705 patients with Medicaid or commercial insurance (see S2 Table) compared with 207,927 unique patients we identified in the APCD with at least one FQHC visit in 2010. Potential reasons for this differential could be incomplete capture of visits in the APCD due to the redaction of visits with substance use diagnoses. To account for this, we adjusted for FQHC-level information from the UDS on the number of patients with SUD-diagnoses; findings were similar. We could also overestimate the total number of people with FQHC visits using the UDS because these data are reported by FQHCs, and we cannot determine if the same patient visited multiple FQHCs. There could also be changes in individual-level insurance status within a year that introduce noise into the UDS estimates.

The claims data have limited information on outpatient visit complexity because Medicaid pays FQHCs an all-inclusive rate such that we could not examine changes in the intensity of visits provided, only the total number of visits. We did not have sufficient power for a sub-analysis in patients with chronic conditions, and future research should investigate the potentially differential effects of funding in that subgroup and others, such as patients in rural areas. We did not correct for multiple comparisons to reduce the likelihood of Type II error [31, 32]; however, in sensitivity analyses we applied the Bonferroni correction to reduce Type I error associated with testing multiple hypotheses across the four primary outcomes such that the family-wise error rate was p = 0.0125 and our findings did not change (see S3 Table for corrected p-values) [33]. This study is specific to Massachusetts and the generalizability of our findings to other states could be limited. Massachusetts had a much lower uninsured rate compared with the rest of the country over this time period (e.g., 4% in Massachusetts vs. 16% nationally in 2010). In addition, because Massachusetts implemented health reform starting in 2006, our findings apply to a state that did not undergo a large insurance coverage expansion as FQHC funding was increasing. Lastly, fluctuations in federal funding in Massachusetts were larger over time compared with national changes, which could reflect the receipt of some larger or special types of grants (e.g., New Access Points) or other regional differences in funding.

## Conclusions

Since 2015, the end of the ACA’s 5-year authorization period for the CHCF, FQHCs have faced potential drop-offs in funding almost every year, with Congress making short-term extensions at the edge of each funding cliff. Many FQHCs reported considering a number of responses to funding delays, e.g., hiring freezes, delays in planned expansions or improvements, and reductions in staff [34]. Such responses could reduce the capacity of FQHCs to serve patients; our study suggests such reductions could have further downstream impacts on ED use and costs. Funding for FQHCs represent a direct public investment in safety net providers, and such investment could be a policy tool to meet growing demand for outpatient care and to reduce costly emergency department care for underserved populations.

## Supporting information

S1 Fig Distributions of shift-share estimated yearly change in FQHC funding in ZIP code. (DOCX)

S2 Fig Unique Medicaid patient count at FQHCs according to APCD claims versus the UDS, 2009–2013. (DOCX)

S3 Fig Emergency department (ED) visit falsification test (future year funding change and changes in ED visits). (DOCX)

S4 Fig Sensitivity analysis using 0.50 threshold for nonemergent and emergent visits in measuring association between changes in total funding and (a) APCD enrollees with ED visits, and (b) ED visits.
(DOCX)

S5 Fig Association between changes in total funding and (a) FQHC visits, and (b) ED visits.
(DOCX)

S1 Table 2010–11 percent (%) change in FQHC-level total funding regressed on 2009 FQHC patient insurance mix, race, income, and age. (DOCX)

S2 Table Characteristics of FQHC patients in the UDS, 2010–2013. (DOCX)

S3 Table Sensitivity analyses including Bonferroni correction for multiple comparisons. (DOCX)

S1 Appendix Constructing shift-share funding predictions. (DOCX)
