# Supplementary material for: The impact of funding for federally qualified health centers on utilization and emergency department visits in Massachusetts
Source: PLoS One. 2020 Dec 3;15(12):e0243279. doi: 10.1371/journal.pone.0243279 (PMC7714363; doi:10.1371/journal.pone.0243279)
Supplement: S1 Fig — (DOCX) [file pone.0243279.s001.docx]

**S1 Fig**. **Distributions of shift-share estimated yearly change in FQHC funding in ZIP code** (n = 559 Massachusetts ZIP codes).

1.
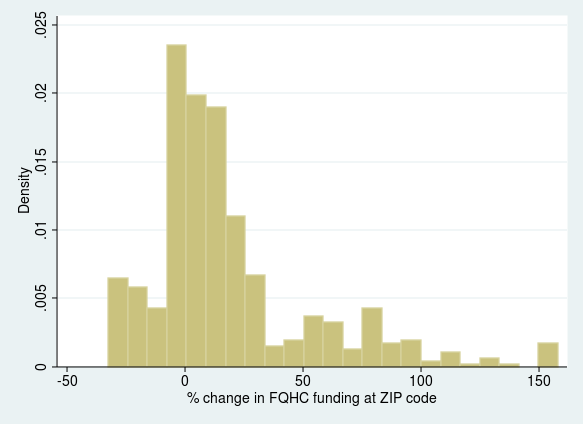

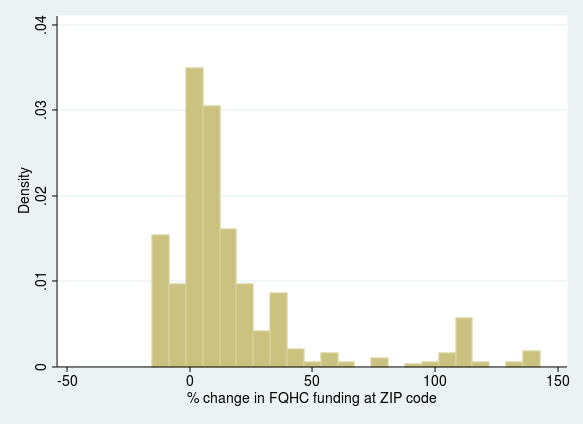
2011 vs. 2010 (mean = 17.8, SD = 31.8) b. 2012 vs. 2011 (mean = 18.7, SD = 36.0)

c. 2013 vs. 2012 (mean = -8.5, SD = 17.4)


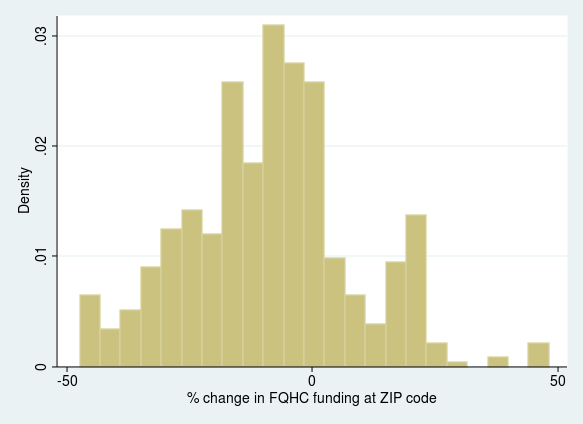


Notes: Data were included when they had at least one visit in APCD 2010-13 and at least one visit in UDS 2010-13.
