# Supplementary material for: The impact of funding for federally qualified health centers on utilization and emergency department visits in Massachusetts
Source: PLoS One. 2020 Dec 3;15(12):e0243279. doi: 10.1371/journal.pone.0243279 (PMC7714363; doi:10.1371/journal.pone.0243279)
Supplement: S2 Fig — (DOCX) [file pone.0243279.s002.docx]

**S2 Fig.** **Unique Medicaid patient count at FQHCs according to APCD claims versus the UDS, 2009-2013** (n = 31 FQHCs).
