# Supplementary material for: The impact of funding for federally qualified health centers on utilization and emergency department visits in Massachusetts
Source: PLoS One. 2020 Dec 3;15(12):e0243279. doi: 10.1371/journal.pone.0243279 (PMC7714363; doi:10.1371/journal.pone.0243279)
Supplement: S3 Fig — (DOCX) [file pone.0243279.s003.docx]

**S3 Fig. Emergency department (ED) visit falsification test (future year funding change and changes in ED visits).**

Notes: Bars indicate 95% confidence interval.
