# Supplementary material for: The impact of funding for federally qualified health centers on utilization and emergency department visits in Massachusetts
Source: PLoS One. 2020 Dec 3;15(12):e0243279. doi: 10.1371/journal.pone.0243279 (PMC7714363; doi:10.1371/journal.pone.0243279)
Supplement: S4 Fig — Sensitivity analysis using 0.50 threshold for nonemergent and emergent visits in measuring association between changes in total funding and (a) APCD enrollees with ED visits, and (b) ED visits. (DOCX) [file pone.0243279.s004.docx]

**S4 Fig: Sensitivity analysis using 0.50 threshold for nonemergent and emergent visits in measuring association between changes in total funding and (a) APCD enrollees with ED visits, and (b) ED visits**.

Notes: Bars indicate 95% confidence interval.
