# Supplementary material for: The impact of funding for federally qualified health centers on utilization and emergency department visits in Massachusetts
Source: PLoS One. 2020 Dec 3;15(12):e0243279. doi: 10.1371/journal.pone.0243279 (PMC7714363; doi:10.1371/journal.pone.0243279)
Supplement: S5 Fig — Association between changes in total funding and (a) FQHC visits, and (b) ED visits. (DOCX) [file pone.0243279.s005.docx]

**S5 Fig.** **Association between Changes in Total Funding and (a) FQHC visits, and (b) ED visits**
