# Supplementary material for: The impact of funding for federally qualified health centers on utilization and emergency department visits in Massachusetts
Source: PLoS One. 2020 Dec 3;15(12):e0243279. doi: 10.1371/journal.pone.0243279 (PMC7714363; doi:10.1371/journal.pone.0243279)
Supplement: S1 Table — (DOCX) [file pone.0243279.s006.docx]

**S1 Table.** **2010-11 Percent (%) change in FQHC-level total funding regressed on 2009 FQHC patient insurance mix, race, income, and age.** (n = 31 FQHCs)

|  | **Estimate** | **95% CI** | |
| --- | --- | --- | --- |
| **Insurance type** |  |  |  |
| % Patient with CHIP | -0.69 | -9.3 | 7.9 |
| Medicare | -0.90 | -5.1 | 3.3 |
| Other public (e.g. Commonwealth Care) | -0.29 | -2.4 | 1.9 |
| Private | 0.08 | -0.8 | 1.0 |
| Uninsured | 0.50 | -1.5 | 2.5 |
| Medicaid (Reference group) | 25.3 | -60.6 | 111.2 |
| **Race** |  |  |  |
| % American Indian | -6.26 | -33.8 | 21.3 |
| Asian | -0.16 | -0.8 | 0.5 |
| Black | 0.27 | -0.8 | 1.4 |
| Hispanic | -0.19 | -0.8 | 0.4 |
| More than one race | -4.24 | -16.7 | 8.2 |
| Unreported | -0.35 | -1.8 | 1.1 |
| White (Reference group) | 25.0 | -27.1 | 77.1 |
| **Income** |  |  |  |
| % Income < 100% FPL | 0.07 | -0.7 | 0.8 |
| 100-150% FPL | -0.09 | -4.4 | 4.2 |
| 150-200% FPL | 0.53 | -4.4 | 5.5 |
| Unknown income | -0.02 | -0.7 | 0.7 |
| Income > 200% FPL (Reference group) | 20.0 | -38.0 | 78.0 |
| **Age** |  |  |  |
| % under age 18 | 1.75 | -1.5 | 5.0 |
| Ages 19-39 | 1.17 | -1.7 | 4.0 |
| Over age 65 | 1.01 | -4.9 | 6.9 |
| Ages 40-65 (Reference group) | -66.7 | -279.6 | 146.1 |
