# Supplementary material for: The impact of funding for federally qualified health centers on utilization and emergency department visits in Massachusetts
Source: PLoS One. 2020 Dec 3;15(12):e0243279. doi: 10.1371/journal.pone.0243279 (PMC7714363; doi:10.1371/journal.pone.0243279)
Supplement: S2 Table — (DOCX) [file pone.0243279.s007.docx]

**S2 Table.** **Characteristics of FQHC patients in the UDS, 2010-2013.**

|  | **UDS-2010** | **UDS-2011** | **UDS-2012** | **UDS-2013** |
| --- | --- | --- | --- | --- |
| **Total N patients** | 561,721 | 589,348 | 612,637 | 634,420 |
| **Age categories** |  |  |  |  |
| <5 | 7.1% | 6.8% | 6.7% | 6.5% |
| 5-17 | 17.0% | 16.3% | 16.2% | 16.0% |
| 18-29 | 21.0% | 20.9% | 20.0% | 20.0% |
| 30-44 | 22.9% | 22.9% | 22.6% | 22.7% |
| 45-64 | 24.2% | 25.1% | 26.0% | 26.0% |
| 65+ | 7.7% | 8.0% | 8.6% | 8.8% |
| **Gender:** Female | 56.0% | 55.9% | 56.1% | 55.9% |
| **Race** |  |  |  |  |
| American Indian/Alaska Native | 0.3% | 0.2% | 0.3% | 0.3% |
| Asian | 9.1% | 9.2% | 9.5% | 9.7% |
| Black | 15.8% | 15.4% | 15.0% | 14.9% |
| Hispanic/Latino | 31.3% | 31.6% | 31.8% | 32.4% |
| Native Hawaiian/ Pacific Islander | 0.3% | 0.2% | 0.2% | 0.3% |
| White | 32.4% | 33.1% | 33.7% | 32.9% |
| Other | 0.6% | 0.6% | 1.0% | 1.9% |
| Unknown/missing | 4.6% | 3.8% | 4.3% | 3.4% |
| **Insurance type (Jan)** |  |  |  |  |
| Commercial total | 19.9% | 20.0% | 19.9% | 20.3% |
| Medicaid total | 42.0% | 41.3% | 41.2% | 40.9% |
| Medicare total | 8.7% | 9.2% | 9.3% | 9.6% |
| Other public | 9.2% | 8.2% | 9.8% | 10.7% |
| Uninsured | 20.2% | 21.3% | 19.7% | 18.4% |
| **Diagnoses received** |  |  |  |  |
| Alcohol related disorders | 0.5% | 0.5% | 1.2% | 1.4% |
| Other SUD (excluding tobacco) | 1.1% | 1.0% | 2.1% | 2.4% |
| Depression & other mood disorders | 4.7% | 4.8% | 8.3% | 9.3% |
| Anxiety disorders | 2.6% | 3.0% | 5.4% | 6.2% |
| Attention deficit | 0.9% | 1.1% | 1.6% | 1.8% |
| Other mental disorders | 2.6% | 3.0% | 4.4% | 5.0% |
