# Supplementary material for: The impact of funding for federally qualified health centers on utilization and emergency department visits in Massachusetts
Source: PLoS One. 2020 Dec 3;15(12):e0243279. doi: 10.1371/journal.pone.0243279 (PMC7714363; doi:10.1371/journal.pone.0243279)
Supplement: S3 Table — (DOCX) [file pone.0243279.s008.docx]

**S3 Table. Sensitivity analyses including Bonferroni correction for multiple comparisons**

| Outcome | Predictor | Coefficient | Corrected p-value |
| --- | --- | --- | --- |
| %Chg in # of enrollees with FQHC visits | current yr | 1.17 | 0.132 |
|  | 1 yr prior | **2.26** | **0.004** |
|  | 2 yrs prior | **1.79** | **0.007** |
| %Chg in # of enrollees with ED visits | current yr | **-1.02** | **0** |
|  | 1 yr prior | -0.65 | 0.114 |
|  | 2 yrs prior | **-0.66** | **0.007** |
| %Chg in # of enrollees with non-emergent ED visits | current yr | **-1.40** | **0** |
|  | 1 yr prior | **-1.32** | **0.01** |
|  | 2 yrs prior | **-1.09** | **0** |
| %Chg in # of enrollees with emergent ED visits | current yr | -0.21 | 0.666 |
|  | 1 yr prior | 0.30 | 0.604 |
|  | 2 yrs prior | -0.16 | 0.753 |
