# Supplementary material for: The impact of funding for federally qualified health centers on utilization and emergency department visits in Massachusetts
Source: PLoS One. 2020 Dec 3;15(12):e0243279. doi: 10.1371/journal.pone.0243279 (PMC7714363; doi:10.1371/journal.pone.0243279)
Supplement: S1 Appendix — (DOCX) [file pone.0243279.s009.docx]

**S1 Appendix:** **Constructing shift-share funding predictions**

Let $x_{jkt}$ represent the amount of funding from FQHC *k* to patients in ZIP code *j* in year *t*. The total amount of funding across all FQHCs affecting ZIP code *j* in this year is $X_{jt}=\sum_{k} x_{jkt}$. The gross percentage change in FQHC funding over two years (say, *t*=0 to *t*=1) can then be written:

$\frac{X_{j1}}{X_{j0}}$=$\sum_{k} \frac{x_{jk1}}{X_{j0}}$=$\sum_{k} \left( \frac{x_{jk0}}{X_{j0}} \right)\left( \frac{x_{jk1}}{x_{jk0}} \right)$

That is, the percentage change in FQHC funding for ZIP *j* can be written in terms of the average change in funding at each of FQHCs that serve ZIP *j* ($x_{jk1}/x_{jk0})$, weighted by each FQHC’s initial share of total funding in ZIP *j* ($x_{jk0}/X_{j0})$.

FQHCs could plausibly direct more resources to neighborhoods with more unobserved need, such that the local funding shocks $x_{jk1}/x_{jk0}$ are higher in ZIP codes facing higher demand. To avoid such reverse causality, the shift-share prediction of local funding changes replaces $x_{jk1}/x_{jk0}$ with $g_{k1}/g_{k0}$ in the above expression, where $g_{kt}$ is the amount of total funding at FQHC *k* in year *t*. As shown in Borusyak et al. (2018), the resulting shift-share instrument can be used to estimate local funding effects, provided that the FQHCs which receive more funding at the national level are not systematically concentrated in regions with higher unobserved demand.

In our setting $x_{jk0}/X_{j0}$, or FQHC *k*’s share of total FQHC funding in ZIP *j*, is not directly observed and must be estimated. We obtain ZIP code patient counts from the UDS, and assume that in the baseline year FQHCs fund allocation is proportional to the distribution of patients, such that: $x_{jk0}=c_{jk0}g_{k0}$

where $c_{jk0}$ is the proportion of FQHC *k*‘s patients from ZIP code *j*. From this we can rewrite the shift-share prediction as

$\frac{X_{j1}}{X_{j0}}$=$\sum_{k} \left( \frac{c_{jk0}g_{k0}}{\sum_{k} c_{jk0}g_{k0}} \right)\left( \frac{g_{k1}}{g_{k0}} \right)$.

This measure averages FQHC-level funding shocks by an exposure measure approximating the FQHC’s share of the total funding dollars in ZIP code *j*. If our underlying assumption for the exposure weights is incorrect, this approach can still estimate causal effects, provided the assumption holds that changes in funding across FQHCs are quasi-randomly distributed from year-to-year. Again, in the framework of Borusyak et al. (2018), quasi-random variation in national funding shocks make this measure appropriate for estimating local funding effects.

In practice, we measure a region’s exposure to FQHC funding (the first term in the above expression) in 2009, and compute national funding changes for each year in 2010-2013. While fixing a lagged exposure measure is not, in general, necessary for quasi-experimental shift-share designs, Borusyak et al. (2018) show how this improves plausibility in panel settings.

Fig 1 in the main text of the manuscript shows a map of MA ZIP codes and their 2010-11 exposure to FQHC funding changes (shift-share instrument) by quantile, with the location of each of the 31 FQHCs included in the study and their 2010-11 percentage change in funding by quantile to illustrate our approach.
